# Public health interventions in midwifery: a systematic review of systematic reviews

**DOI:** 10.1186/1471-2458-12-955

**Published:** 2012-11-08

**Authors:** Jenny McNeill, Fiona Lynn, Fiona Alderdice

**Affiliations:** 1 School of Nursing & Midwifery, Queen’s University Belfast, Medical Biology Centre, 97 Lisburn Road, Belfast, BT9 7BL, Northern Ireland

**Keywords:** Systematic review, Public health, Midwife, Pregnancy

## Abstract

**Background:**

Maternity care providers, particularly midwives, have a window of opportunity to influence pregnant women about positive health choices. This aim of this paper is to identify evidence of effective public health interventions from good quality systematic reviews that could be conducted by midwives.

**Methods:**

Relevant databases including MEDLINE, Pubmed, EBSCO, CRD, MIDIRS, Web of Science, The Cochrane Library and Econlit were searched to identify systematic reviews in October 2010. Quality assessment of all reviews was conducted.

**Results:**

Thirty-six good quality systematic reviews were identified which reported on effective interventions. The reviews were conducted on a diverse range of interventions across the reproductive continuum and were categorised under: screening; supplementation; support; education; mental health; birthing environment; clinical care in labour and breast feeding. The scope and strength of the review findings are discussed in relation to current practice. A logic model was developed to provide an overarching framework of midwifery public health roles to inform research policy and practice.

**Conclusions:**

This review provides a broad scope of high quality systematic review evidence and definitively highlights the challenge of knowledge transfer from research into practice. The review also identified gaps in knowledge around the impact of core midwifery practice on public health outcomes and the value of this contribution. This review provides evidence for researchers and funders as to the gaps in current knowledge and should be used to inform the strategic direction of the role of midwifery in public health in policy and practice.

## Background

The reproductive period offers maternity care providers the opportunity to maximise the health and well-being of women and their families potentially impacting on public health outcomes, both short and long term. Although all maternity care providers who engage with pregnant women are presented with such opportunities, it is the midwife that could have the most significant impact from regular contact and building of relationships through continuity of care. There are interventions that could be implemented by midwives, which potentially would have a public health impact but it is important such interventions are evidence based. Recognition of the importance of the relationship between public health and midwifery was highlighted when a general review of midwifery in the UK [1], named public health as one of five key areas of interest. While the review specifically focused on midwifery in the UK, the importance of preventative public health interventions during pregnancy and the postnatal period has been emphasized on a wider scale. Millennium Development Goal 5 focuses on improving maternal health specifying a secondary target aim to achieve universal access to reproductive health by 2015 [2]. Antenatal care and adolescent pregnancy are specifically mentioned as key to achieving this target, both of which are acknowledged widely, as areas of interest to public health [3,4]. Other areas of national and international interest, which impact on population health (both women and families), include rising caesarean section rates and other interventions during childbirth [5-7], the importance of positive parenting in the early postnatal period [8] and perinatal mental health [9]. Within these areas there is opportunity for evidence based public health interventions to be implemented with a view to potentially improving the long term health of women and families.

### Aim of the review

This paper presents an update of a systematic review of systematic reviews conducted in 2009. The aim of the 2009 review was to evaluate the effectiveness of interventions relevant to the public health role of the midwife. The 2009 review was commissioned and conducted within the context of the Midwifery 2020 initiative. The final report of the Midwifery 2020 initiative (Delivering Expectations) and full report of the systematic review of reviews [10] are available freely online from: http://www.midwifery2020.org. A systematic review of systematic reviews was selected as the methodology, given the breadth of this topic area and the timescale of the project. This paper outlines the review methodology and builds on the original review findings by providing new and updated information about effective high quality public health interventions which could be implemented by midwives or other health care providers for women during pregnancy and the postnatal period who have a similar role, for example, public health nurses, obstetric nurses, labour and delivery nurses or health visitors.

## Methods

The Preferred Reporting Items of Systematic reviews Meta-Analyses (PRISMA) guidelines was adhered to when conducting this review [11]. A systematic search strategy was formulated and definitive search terms used relative to key public health topics within midwifery following consultation with Expert Advisory Group members and Midwifery 2020 Public Health Work Stream members. Seven key areas were identified as relevant to the public health role of the midwife, which included: screening; vulnerable groups; breast feeding; mental health and wellbeing; education and support; childbirth and lifestyle factors. The complete list of search terms is available from McNeill *et al.*[10].

### Search strategy

Databases searched included: MEDLINE, PubMed, EBSCO (CINAHL/British Nursing Index), MIDIRS Online Database, Web of Science, The Cochrane Library, CRD (NHS EED/DARE/HTA) and EconLit. Eligibility criterion included reviews published from 1999 onwards; English language publications and reviews originating from economically developed countries as indicated by membership of the Organisation for Economic Co-operation and Development (OECD). An additional search was conducted of the National Institute for Health and Clinical Excellence, UK (NICE) website to identify key publications or findings from systematic reviews within guidelines. Reference lists of identified reviews were manually searched for additional relevant reviews. The searches were initially conducted in November 2009 and updated in October 2010. The titles and abstracts were obtained and the decision process for eligibility was conducted by all members of the project team in collaboration (JM, FL & FA). Full text was obtained of all eligible reviews and those whose eligibility could not be discerned from reading the abstract. Eligible systematic reviews also had to publish a clearly identified search strategy or detail the reference databases used.

### Data extraction

Data were extracted on: number of papers included in the review; methodological details; midwifery intervention; outcome measures and results. Data were systematically extracted using a data extraction form by individual project team members and verified by one other project team member. The project team subsequently met to discuss and achieve consensus regarding any contentious issues. A parallel process of developing a logic model to act as an overarching framework to inform forward planning was also conducted. Logic models are essentially a conceptual framework, which can be used for evidence‐based decision making and planning [12]. The model is composed of midwifery inputs and activities, producing a logical pathways to short, medium and long term public health outputs.

#### Quality assessment and effectiveness of reviews

It is important to consider both the type of evidence included in reviews i.e. was the review restricted to randomised trials only or were other types of studies included and also assess how well the review was conducted methodologically. As such, a two stage process was employed: initially the level of evidence was graded and secondly, the methodological quality was assessed. Recognised frameworks were used to support this process [13,14]. In the hierarchy of evidence, randomised controlled trials are perceived as the gold standard and as the aim of this paper is to present high quality evidence, an evidence grade was given to each review based on the Scottish Intercollegiate Guidelines Network [13] framework in order to distinguish between different levels of evidence. This framework grades the associated risk of bias based on the level of evidence in a hierarchal manner from a grade of 1++ (meta analysis and RCT evidence) through to 4 (expert opinion), as outlined in Table 1. The SIGN framework was modified as this review was restricted to systematic reviews and therefore reviews could only be graded as 1++, 1+, 1- or 2++. This paper only presents evidence which was graded 1- or above; any review graded below 1- was not deemed eligible for inclusion. Following selection of the type of evidence, the second stage focused on the methodology of eligible reviews. Clarke [15] suggests the successful interpretation of results from systematic reviews should consider the methodological conduct of the review. The methodological quality of included reviews was assessed and rated as low, medium or high quality. Appraisal of methodological quality was based on Smith *et al.*[14], which contains similar elements to other tools used to assess review quality, for example, the AMSTAR tool [16]. Reviews were graded as high quality if they included evidence of a search strategy, selection and inclusion criterion, assessment of publication bias and assessment of heterogeneity. Reviews were rated as medium quality if no evidence of assessment of heterogeneity or publication bias was provided and low quality reviews were those which provided evidence of a search strategy only. Effectiveness of interventions was evaluated using a similar approach to van Sluijs *et al.*[17]. A differentiation was made between reviews which reported a statistically significant difference (P<0.05), therefore referred to as effective and those which reported no difference in effect between control and intervention group and are referred to as inconclusive or not effective (as appropriate). This paper focuses specifically on interventions which are evidenced by a statistically significant meta analysis or where the intervention is supported by a generally positive trend of results when a meta analysis was not possible. Reviews have been included where a small number of studies reported statistically significant positive effect of the intervention however the wider interpretation of these results is limited. As outlined previously, the aim of the original review was to identify *any* public health intervention relevant to midwifery. However for the purpose of this paper the focus was to report on public health interventions relating to midwifery that demonstrated a statistically significant effect in favour of the intervention (referred to subsequently as effective interventions for the sake of brevity). Reviews graded 1- or above and of high methodological quality which reported evidence of no effect, are not discussed in this paper. However, they have been summarised in Table 2[18-23]. In the case of any disagreement regarding grading of evidence, quality appraisal of reviews or effectiveness of the intervention, consensus was reached by discussion between all three authors.

**Table 1:** Evidence level of systematic reviews

**Score**	**Source of evidence**
1++	High quality meta analyses or systematic reviews of RCT’s
1+	Well conducted meta analyses or systematic reviews of RCT’s
1-	Meta analyses or systematic reviews of RCT’s
2++	High quality systematic reviews of case control or cohort studies
2+	Well conducted case control or cohort studies with low risk of bias
2-	Case control or cohort studies with high risk of bias
3	Case reports or case series
4	Expert opinion or formal consensus

From Scottish Intercollegiate Guidelines Network [13].

**Table 2:** Excluded High Quality Reviews Reporting Interventions with no effect

**Author & Year**	**Number of papers included (date range)**	**Intervention**	**Key outcomes of Interest**	**Key Findings**
Bricker *et al*. [18]	8 (1984-2003)	Routine USS in pregnancy after 24weeks	Primary: induction of labour, caesarean section, all deaths, preterm delivery <34weeks, neurodevelopment at age 2yrs & maternal psychological effects	No difference in antenatal, obstetric and neonatal intervention or morbidity in groups
			Secondary: interventions, additional maternal, perinatal and neonatal outcomes)	Routine USS not associated with improved perinatal mortality Increased Caesarean rate in screened group-non significant (RR 1.06 95% CI 1.00 -1.13, *p* = 0.07)
Carrolli *et al*. [19]	7 (1995-2001)	Routine antenatal care patterns	Effect of reduced number of visits v standard number of visits on: Pre-eclampsia, UTI, postpartum anaemia, maternal mortality, LBW and perinatal mortality	Pre-eclampsia: no difference (OR 0.91 95% CI 0.66-1.26)
				UTI: no difference (OR 0.93 95% CI 0.79-1.10)
				Postpartum anaemia: no difference (OR 1.01)
				Maternal mortality :no difference (OR 0.91 95% CI 0.55-1.51)
				LBW: no difference (OR 1.04 95% CI 0.93-1.17)
				Perinatal mortality: rates similar although rare outcome so no statistical equivalence
				Some dissatisfaction of women with care and fewer visits
Grivell et al. [20]	6 (1982-1999)	Cochrane: Antenatal CTG for fetal assessment	Primary: perinatal mortality and CS	Comparison of traditional CTG versus no CTG showed no significant difference identified in perinatal mortality (RR 2.05, 95% CI 0.95 to 4.42, 2.3% versus 1.1%, four studies, N = 1627)
			Secondary: potentially preventable perinatal mortality (exc lethal congenital anomalies), Apgar < 7 @ 5mins, Apgar < 4@ 5mins, Cord pH < 7.10 or low pH/low base excess, Admission to NICU/ICU, Length of stay in neonatal SCU or ICU, Preterm birth (< 37 completed weeks, <34 completed weeks, <28 completed weeks), Gestational age at birth	No significant difference identified in caesarean sections (RR 1.06, 95% CI 0.88 to 1.28, 19.7% versus 18.5%, three trials, N = 1279) nor in the secondary outcomes that were assessed.
			Neonatal seizures, Hypoxic ischaemic encephalopathy, Cerebral palsy at 12 months, neurodevelopmental disability at more than 12 months, CS non-reassuring or abnormal FHR, IOL , antenatal hospital admission, length of antenatal hospital stay, emotional distress, depression, anxiety and satisfaction with care	
Kongnyuy *et al*. [21]	5 (1999-2006)	Provision of advice regarding vitamin A supplementation in HIV infected women	Risk of Mother-to-Child Transmission (MTCT) of HIV,birth weight, stillbirth rate and PTD	No evidence of an effect on the risk of prenatal or postnatal MTCT of HIV (RR 1.06, 95% CI 0.89-1.26). Prenatal vitamin A improved infant birth weight (WMD 89.78, 95% CI 84.73-94.83), but had no effect on stillbirth rate (RR 0.99, 95% CI 0.68-1.43) or PTD (RR 0.88, 95% CI 0.65-1.19).
Rumbold *et al*. [22]	10 (1994-2006)	Antioxidant supplementation for preventing pre-eclampsia	Pre-eclampsia, severe pre-eclampsia, preterm birth, SGA infants, infant death	No significant difference for pre-eclampsia or any other primary outcome-does not support routine antioxidant supplementation to reduce risk of pre-eclampsia
Villar *et al*. [23]	10 (1992-2001)	Provision of antenatal care for low risk pregnancy-reduced number of visits	Preterm delivery, pre-eclampsia, anaemia, urinary tract infection, CS, IOL, APH, PPH, LBW, SGA, perinatal mortality, maternal mortality, cost effectiveness and perception of care	No difference in any outcomes
				Women in developed countries are more likely to be less satisfied with with fewer visits
				Antenatal care provided by a midwife/general practitioner was associated with improved perception of care by women

Acronyms used: USS=Ultrasound Scan; CI=confidence interval; RR=relative risk; UTI=urinary tract infection; OR=odds ratio; LBW=low birth weight; CS=caesarean section; NICU=neonatal intensive care unit; ICU=intensive care unit; SCU=special care unit; FHR=fetal heart rate; IOL=induction of labour; CTG=cardiotocography; PTD=preterm delivery; WMD=weighted mean difference; APH=antepartum haemorrhage; PPH=postpartum haemorrhage; SGA=small for gestational age.

### Data synthesis

A narrative review is provided for each of the systematic reviews and in table format the number and date range of papers included, intervention(s), primary outcome or other public health outcomes of interest, results (including key statistical findings e.g. p values or odds ratios) are described and whether the review included a meta analysis or not. It was not expected that a quantitative analyses would be conducted given the diversity of interventions across the broad subject of public health.

## Results

**Figure 1:**
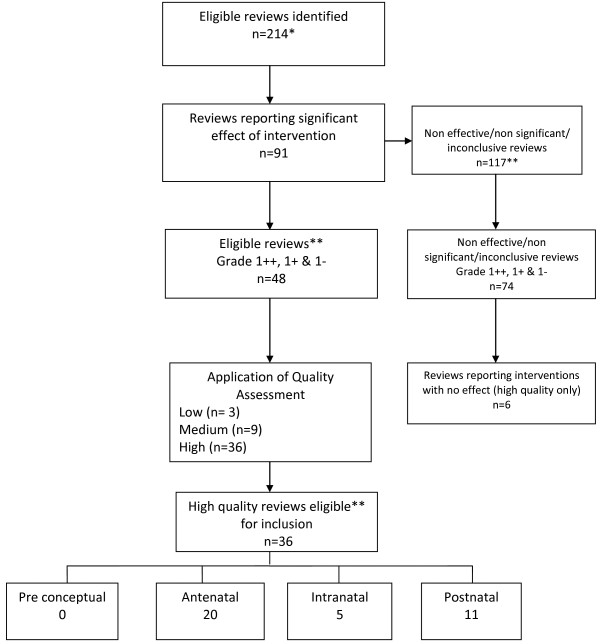
Identification of effective reviews of high quality *some reviews which were included at the request of funder have been excluded from this paper eg economic reviews (n=6) **non significant, non effective or inconclusive reviews, reviews graded 2++,2+ or 2- and medium or low quality reviews are not discussed in this paper.

In total 214 systematic reviews were eligible of which 91 reported on effective interventions and 117 found no effect or were inconclusive. This paper only reports on high quality reviews with a level of evidence grading above 1-. Of the 91 systematic reviews which reported on effective interventions, 36 were identified which were graded as evidence level 1- or above and rated as high quality. The flow chart in Figure 1 presents the sequential process of identifying reviews eligible for inclusion in this paper. An overview of the key findings in relation to interventions demonstrating a statistically significant effect in favour of the intervention from good quality reviews will be presented in the following sections. A summary of included reviews is provided in Table 3. The findings in this paper are presented chronologically through the reproductive period: preconceptual; antenatal; intranatal and postnatal. Within each section the reviews on similar broad topics have been further categorised: antenatal (screening; supplementation; support; education; mental health); intranatal (clinical care; environment); postnatal (breast feeding; mental health; education; support). The findings section also presents the logic model which was developed in parallel with the searching and analysis of reviews. Logic models enable the visualisation of how interventions or programmes work and the expected outcomes [24] and have been used to consider the strategic public health benefit of midwifery practice both in the short and long term [25].

**Table 3:** Included Reviews

**Author & Year**	**Number of papers included (date range)**	**Intervention**	**Primary outcome(s) and additional key public health outcomes of interest**	**Main Results /Findings**	**Meta Analysis**
***Antenatal***					
***Screening***					
Bricker *et al.*[26]	79 (1980-1998)	Routine USS in pregnancy	1. Clinical effectiveness of USS (11)	1. 2 stage regimen of USS in pregnancy recommended (early & around 20 weeks)	No
			2. Cost effectiveness of USS (9)	2. cost effectiveness results not reported on in this paper	
			3. Women’s views of USS (59 studies, 76 reports)	3. USS is attractive to women. Findings of uncertain clinical significance may impact psychologically on women-further research needed. Anxiety reduction after USS is likely to reflect raised anxiety prior to scan than a real reduction	
Whitworth *et al.*[27]	11 (1982-2009)	Routine USS in early pregnancy	Primary: detection of major fetal abnormality, detection of multiple pregnancy, Induction of labour for ’post-term’ pregnancy, perinatal death Multiple secondary outcomes	Reduces failure to detect multiple pregnancy (RR 0.07 95% CI 0.03-0.17)	Yes
				Reduction in IOL for post term (RR 0.59 95% CI 0.42-0.83)	
Sangkomkam-hang *et al*. [28]	1 (2004)	Antenatal lower genital tract infection screening to prevent PTD	Primary: preterm birth	One trial (n=4155) was included. Preterm birth was significantly lower in the intervention group than in the control (RR 0.55 95% CI 0.41-0.75).	No
			Secondary: LBW, very LBW, neonatal morbidity, duration of admission to NICU or hospital, death, treatment side effects, persistent infection, recurrent infection, failure of treatment, economic analysis, false positive/negative result of screening program and women’s satisfaction	Preterm birth for LBW (RR 0.48 95% CI 0.34-0.66) and very LBW (RR 0.34 95% CI 1.5-0.75) was significantly lower in the intervention group than in the control group.	
O’Connor *et al.*[29]	55 (1983-2006)	The use of decision making aids for people facing difficult screening decisions	Use of decision aids when providing information about screening	The use of decision aids are better than usual care in relation to knowledge (MD 15.2 out of 100 95% CI 11.7 to 18.7), decisional conflict related to feeling uninformed (MD -8.3 of 100; 95% CI -11.9 to -4.8), risk perceptions and reduced passive involvement in decisions (RR 0.6; 95% CI 0.5 to 0.8)	Yes
***Supplementation***					
Blencowe *et al.*[30]	19	Folic acid supplementation	Neonatal mortality from NTD	FA supplementation in women with previous pregnancy with NTD: 70% reduction (95% CI 35-86) (3xRCT)	Yes
Lumley *et al.*[31]	4 (1981-1999)	Periconceptual supplementation with folate/multivitamins	NTD incidence	↓incidence of NTD (RR 0.28, 95% CI 0.13 to 0.58)	Yes
Pena-Rosas & Viteri [32]	49 (1958-2008)	Iron supplementation in pregnancy	Maternal: premature delivery, Hb at term, anaemia at term, iron deficiency at term, iron-deficiency anaemia at term, side effects Infant: LBW, birthweight	Daily iron supplementation associated with ↑ Hb at term (MD 6.00 95% CI 2.75-9.25, high quality trials only included in MA) before & after birth and ↓risk of anaemia at term (RR 0.46; 95% CI 0.29 to 0.72, MA from 4 high quality trials)	Yes
Shah *et al.*[33]	13 (1998-2007)	Prenatal micronutrient supplementation	Pregnancy outcome, low birth weight, pre term birth, SGA, birth weight & gestational age	Reduction in risk of LBW (RR 0.81 95%CI 0.73-0.91) compared with placebo or folic acid RR 0.74 95% CI 0.74-0.93). Birth weight higher (WMD 54g 95%CI 36-72g)	Yes
Hofymeyr *et al.*[34]	11 (1987-1999)	Calcium supplementation	Maternal: ↑B/P with or without proteinuria, ↑B/P with significant proteinuria, Infant: preterm delivery, birthweight, admission to NICU, stillbirth or death	Pre-eclampsia ↓(RR 0.68, 95% CI 0.57-0.81)	Yes
				Fewer babies born <2500g (RR 0.83 95% CI 0.71-0.98)	
Hofmeyr *et al.*[35]	12 (1987-2006)	Calcium supplementation	Maternal: ↑B/P with or without proteinuria, ↑B/P with significant proteinuria, maternal death or serious morbidity	B/P ↓ with supplementation (RR 0.7, 95% CI 0.57-0.86)	Yes
				Pre-eclampsia ↓(RR 0.48, 95% CI 0.33-0.69)	
				Maternal death/morbidity ↓(RR 0.80 95% CI 0.65-0.97)	
				Comment from author: possible benefit in research to investigate calcium at community level	
Hofymeyr *et al.*[36]	13 (1987-2009)	Calcium supplementation	Maternal: ↑B/P with or without proteinuria, ↑B/P with significant proteinuria, maternal death or serious morbidity, placental abruption, C/S, proteinuria, severe pre-eclampsia, eclampsia, HELLP syndrome, ICU, maternal death, mother’s hospital stay seven days or more	Risk of ↑B/P reduced (RR 0.65, 95% CI 0.53-0.81)	Yes
			Infant: Preterm birth, LBW, SGA, admission to NICU, neonate in NICU >7days stillbirth or death before discharge from hospital, death or severe neonatal morbidity	Risk of preeclampsia reduced (RR 0.45, 95% CI 0.31-0.65)	
				Effect was greatest for high risk women (RR 0.22, 95% CI 0.12 to 0.42) and women with low baseline calcium (RR 0.36, 95% CI 0.20 to 0.65)	
				Maternal death or serious morbidity reduced (RR 0.80 95% CI 0.65-0.97	
Horvath *et al.*[37]	4 (1995-2000)	Advice regarding polyunsaturated fatty acids supplementation to women in high-risk pregnancies	Duration of pregnancy, preterm delivery (PTD), (birth weight, occurrence of intrauterine growth retardation (IUGR)	Significantly lower rate of PTD <34 wks (RR 0.39, 95% CI 0.18-0.84). (from 2xRCT, n=291)	Yes
***Support***					
Hatem *et al.*[38]	11 (1989-2003)	Midwifery led models of care	1. Antenatal: mean number of antenatal visits, antenatal hospitalisation, APH, fetal loss and neonatal death <24wks, fetal loss or neonatal death ≥ 24wks, total fetal loss and neonatal death	-Women who had midwife-led models of care were less likely to experience antenatal hospitalisation, (RR 0.90 95% CI 0.81-0.99), regional analgesia (RR 0.81, 95%CI 0.73-0.91), episiotomy(RR 0.82, 95% CI 0.77-0.88), and instrumental delivery (RR 0.86, 95% CI 0.78 to 0.96)	Yes
			2. Labour: amniotomy, augmentation/artificial oxytocin during labour, no intrapartum analgesia/ anaesthesia, regional analgesia, opiate analgesia, mean labour length, IOL	-Women were more likely to experience no intrapartum analgesia/anaesthesia (RR 1.16, 95% CI 1.05-1.29), SVB (RR 1.04, 95% CI 1.02-1.06), feeling in control during childbirth (RR 1.74, 95% CI 1.32-2.30), attendance at birth by a known midwife (RR 7.84, 95% CI 4.15-14.81) and initiate breastfeeding (RR 1.35, 95% CI 1.03 to 1.76)	
			3. Delivery and immediate postpartum: C/S, attendance at birth by known carer, instrumental vaginal birth, SVB, episiotomy, perineal laceration requiring suturing, intact perineum, PPH, maternal death, duration of postnatal hospital stay (days)	-No difference between groups for C/S births (RR 0.96, 95% CI 0.87-1.06).	
			4. Neonatal: LBW, preterm birth, Apgar score <7 at 5 mins, admission to SCU, NICU, mean length of neonatal hospital stay, neonatal convulsions, cord blood acidosis, maternal postpartum, postpartum depression, breastfeeding initiation, any breastfeeding at three months, prolonged perineal pain, pain during sexual intercourse, Urinary incontinence, faecal incontinence, prolonged backache, high perceptions of control during labour and childbirth	-Women who were randomised to receive midwife-led care were less likely to experience fetal loss before 24 weeks’ gestation (RR 0.79, 95% CI 0.65-0.97)	
				-No statistically significant differences in fetal loss/neonatal death of at least 24 weeks (RR 1.01, 95% CI 0.67-1.53) or fetal/neonatal death overall (RR 0.83, 95% CI 0.70-1.00) and their babies were more likely to have a shorter length of hospital stay (mean difference -2.00, 95%CI -2.15 to -1.85)	
Hodnett & Fredericks [39]	18 (1986-2001)	Additional emotional support, with information and advice, to women at risk of PTD or delivering a LBW baby	PTD, birth weight (mode of delivery, pregnancy outcome, psychosocial outcomes)	Offering additional emotional support was not associated with improved perinatal outcomes.	Yes
				Associated reduction in caesarean births (RR 0.88, 95% CI 0.79-0.98) and an increase in likelihood of elective termination of pregnancy (RR 2.96, 95% CI 1.42-6.17).	
Dennis & Kingston [40]	14 (1986-2004)	Telephone support during pregnancy and early postpartum	Smoking abstinence, smoking relapse, smoking cessation, preterm birth, LBW, breastfeeding, postpartum depression	Reduction in LBW (3 trials, n=2,027; RR=0.78, 95% CI 0.63-0.97)	Yes
				Improved continuation of any breast feeding (3 trials, n=618; RR=1.18, 95% CI 1.05-1.33) and exclusive breast feeding (2 trials, n=295; RR=1.45, 95% CI 1.12-1.87)	
				Postpartum depression: significant effect at 4 weeks (RR 0.24 95% CI 0.06-1.00) and 8 weeks (RR 0.30 95% CI 0.10-0.92) based on pilot trial data	
				Some methodological limitations of trials included and majority of MA conducted with small number of studies	
***Education***					
Lumley *et al.*[41]	72 (1975-2008)	Promotion of smoking cessation in pregnancy	Smoking cessation, smoking reduction, birth weight, mode of birth, perinatal outcomes, breastfeeding, gestation, psychological measures, withdrawals	Significant reduction in smoking in late pregnancy (RR 0.94, 95% CI 0.93-0.96)	Yes
				Reduction in LBW (RR 0.83, 95% CI 0.73 -0.95) and preterm birth (RR 0.86, 95% CI 0.74-0.98)	
				53.91g (95% CI 10.44 g to 95.38 g) increase in mean birth weight	
Naughton *et al.*[42]	15 (1985-2003)	Self help smoking interventions in pregnancy -written, audio, telephone or computer based	Effectiveness of self help on cessation of smoking	Self help more effective: 13.2% quit rate v 4.9% (OR 1.83 95% CI 1.23-2.73)	Yes
Hay-Smith *et al.*[43]	16 (1987-2007)	Pelvic floor muscle training	Self-reported urinary or faecal incontinence	Women without prior incontinence were less likely to report incontinence in late pregnancy (RR 0.44 95% CI 0.30-0.65) and up to 6 mths postpartum (RR 0.71 95% CI 0.52-0.97)	Yes
				Pregnant women with persistent incontinence 3mths after delivery and received PMFT less likely to report urinary incontinence at 12 mths post delivery (RR 0.79 95% CI 0.70-0.90) and less likely to report faecal incontinence at 12 mths (RR 0.52 95%CI 0.31-0.87)	
Lemos *et al.*[44]	4 (1998-2004)	Perineal exercises during pregnancy	Prevention of urinary incontinence	Significantly reduced development of urinary incontinence from 6 weeks to 3 mths after delivery (OR 0.45 95% CI 0.3-0.66) 4x RCT, n=675	Yes
***Mental Health***					
Dennis & Creedy [45]	15 (1966-04)	Psychosocial and psychological interventions for preventing postpartum depression	Postpartum depression/ psychosis Maternal mortality	Women receiving psychosocial interventions were equally likely to develop depression as those receiving standard care (RR 0.81, CI 0.65-1.02).	Yes
				Identifying mothers at risk assisted prevention of postpartum depression (RR 0.67, CI 0.51-0.89)	
				Interventions with only postnatal component more beneficial than those also incorporating antenatal component (RR 0.76, CI 0.58-0.98)	
				Individually based interventions more effective than group based (RR 0.76, CI 0.59-1.00)	
				No preventive effect of psychological debriefing (RR 0.57 CI, 0.31-1.04)	
***Intranatal***					
***Clinical Care***					
Cluett & Burns [46]	11 (1993-2007) 11xRCT	Immersion in water for labour or birth	Maternal outcomes (mortality, morbidity & labour)	First stage of labour: significant reduction in the epidural/	Yes
			Fetal outcomes (abnormal heart rate, meconium, birth weight & gestational age)	spinal/paracervical analgesia/	
			Neonatal outcomes (morbidity & mortality)	anaesthesia rate amongst women allocated to water immersion compared to controls (478/1254 versus 529/1245; (OR) 0.82, 95% CI 0.70-0.98, p 0.025) 6xRCT	
			Care giver outcomes (satisfaction & injury)		
Rabe *et al.*[47]	7 (1988-2000)	Early umbilical clamping in pre term infants	Requirement for resuscitation	Delayed clamping associated with fewer transfusions for anaemia (RR 2.01, 95% CI 1.24-3.27) 3x RCT, n=111	Yes
			Apgar score at 5 & 10 mins	low B/P (RR 2.58, 95% CI 1.17 to 5.67) 2x RCT, n=58	
			Hypothermia during first hour of life on admission or in labour ward	less IVH (RR 1.74, 95% CI 1.08-2.81) 5xRCT, n=225	
			Death	No difference in other outcomes	
			(B/P, IVH)		
***Environment***					
Hodnett *et al.*[48]	9 (1984-2009)	Alternative v conventional settings for birth	Spontaneous vaginal birth.	Alternative setting increased likelihood of no intrapartum analgesia/anaesthesia (RR 1.17, 95% CI 1.01-1.35), SVB (RR 1.04, 95% CI 1.02-1.06), positive views of care (RR 1.96, 95% CI 1.78-2.15), breastfeeding at 6-8 wks (RR 1.04, 95% CI 1.02-1.06) decreased episiotomy rate (RR 0.83, 95% CI 0.77-0.90)	Yes
			Maternal death or serious maternal morbidity	no effect on serious perinatal or maternal morbidity/mortality	
			Use of analgesia/anaesthesia for labour or birth.		
			Labour augmentation with artificial oxytocics.		
			Views of intrapartum care		
			Perinatal death or serious perinatal morbidity		
Hodnett *et al.*[49]	16 (1989-2006) 16x RCT	Provision of continuous support for women during childbirth	Labour: ARM, oxytocin, EFM, epidural analgesia, any analgesia/anaesthesia, severe pain, labour length.	Women who had continuous intrapartum support were likely to have a slightly shorter labour, (WMD -0.43 hours, 95% CI -0.83 to -0.04), were more likely to have a spontaneous vaginal birth (RR 1.07, 95% CI 1.04 to 1.12) and less likely to have intrapartum analgesia ( RR 0.89, 95% CI 0.82- 0.96) or to report dissatisfaction with their childbirth experiences (RR 0.73, 95% CI 0.65- 0.83)	Yes
			Birth events: C/S, operative vaginal birth, SVB, episiotomy, perineal trauma		
			Newborn events: low 5min APGAR, low cord pH, admission to SCU, prolonged newborn hospital stay		
			Immediate maternal psychological outcomes: feeling tense, anxious during labour, negative rating of/negative feeling about the experience, perceived difficulty in coping with labour, perceived low control during labour		
			Longer-term maternal outcomes: PND, low self-esteem in the postpartum period, anxiety in the postpartum period, difficulty mothering, less than full breastfeeding, prolonged perineal pain, pain during sexual intercourse, urinary incontinence, faecal incontinence;		
Chaillet & Dumont [50]	10 (1992-2005)	Evidence based strategies to reduce C/S rate	Reduction in C/S rate	Significant reduction in all studies of C/S rate (RR 0.81 95% CI 0.75-0.87)	Yes
				Types of strategies effective to reduce C/S rate were audit & feedback (RR 0.87 0.81-0.93), quality improvement (RR 0.74, 95% CI 0.70-0.77) and multifaceted strategies (RR 0.73 95% CI 0.68-0.79)	
				Studies including an identification of the barriers to change were more effective (RR 0.74 95% CI 0.71-0.78) than studies which did not (RR 0.88 95% 0.82-0.96)	
***Postnatal***					
***Breast Feeding***					
Britton *et al.*[51]	34 (1979-2004)	Additional support for breastfeeding mothers	Duration of any breastfeeding (exclusive/ partial breastfeeding)	All forms of additional support resulted in longer duration of any breastfeeding (RR 0.91, 95% CI 0.86-0.96), with the largest effect on exclusive breastfeeding than any (RR 0.81, 95% CI 0.74-0.89).	Yes
				Lay and professional support together extended any breastfeeding significantly (before 4-6wks RR 0.65, 95% CI 0.51-0.82; before 2 mths RR 0.74, 95% CI 0.66-0.83) Exclusive breastfeeding significantly prolonged with WHO/UNICEF training (RR 0.69, 95% CI 0.52-0.91).	
				Face-to-face contact with mothers more useful than telephone contact.	
Chung *et al.*[52]	38 (2001-2007)	Promotion of breastfeeding through education, support or other component	Breastfeeding rates (initiation, duration and exclusivity)	Breastfeeding interventions increased rates of short-term (1-3mths) and long-term (6-8mths) exclusive breastfeeding (RR 1.28, 95% CI 1.11-1.48 and RR 1.44, 95% CI 1.13-1.84) although statistically significant heterogeneity was noted for short term exclusive breast feeding (I^2^ =55%; p= 0.006).	Yes
				Increased rate (22%) of any (RR 1.22 95%CI 1.08-1.37) and exclusive (RR 1.65 95%CI 1.03-2.63) short term breastfeeding with interventions that included a component of lay support.	
				Pre- and postnatal breastfeeding interventions together had a larger effect than either alone.	
				Inclusion of lay support was more effective than usual care in increasing short term rates.	
				No evidence to support formal breastfeeding education with individual level professional support for increasing initiation rates.	
Sikorski *et al.*[53]	20 (1979-2001)	Additional support for breastfeeding mothers vs. standard care	Breastfeeding rates: duration and exclusivity	Additional professional support was more beneficial than standard care for duration of any breastfeeding (RR 0.89, 95% CI 0.81-0.97) 10xRCT, n=19,696 6xRCT, n=18,258	Yes
				Additional lay support was effective in reducing the cessation of exclusive breastfeeding (RR 0.66, 95% CI 0.49-0.89), 5xRCT, n=2530	
				Effect sizes for interventions with a postnatal element alone were (RR 0.80, 95% CI 0.80-0.96).	
				Four trials using WHO/UNICEF training showed significant benefit in prolonging exclusive breastfeeding (RR 0.70, 95% CI 0.53-0.93)	
Dyson *et al.*[54]	11 (1987-2004)	Interventions occurring before the first feed to promote initiation of breastfeeding	Breastfeeding initiation rates	Five studies (n=582) showed breastfeeding education had a significant effect on increasing breastfeeding initiation rates compared to standard care (RR 1.57, 95% CI 1.15-2.15, p=0.005) in low income groups. Substantial statistical heterogeneity noted (I^2^=53.4%)	Yes
				One-to-one, needs-based, informal repeat education sessions and formal antenatal education sessions were effective (2 studies, n=162, RR 2.40, 95% CI 1.57-3.66, Z = 4.05; p= 0.000051) in increasing breastfeeding rates among low income mothers regardless of ethnicity & feeding intention. Statistical heterogeneity small (I^2^=7.0%)	
				One study (n=165) showed needs-based, informal peer support in antenatal and postnatal periods was effective (RR 4.02, 95% CI 2.63-6.14, p<0.00001) in increasing initiation but not seen at 1 or 3 months post partum.	
Moore *et al*[55]	35 (1976-2005)	Early skin to skin contact	Breastfeeding status (exclusivity) and duration	Statistically significant and positive effects of early skin to skin on breastfeeding at one to four months post birth (10 trials; 552 participants (OR 1.82, 95% CI 1.08-3.07) and breastfeeding duration (seven trials; 324 participants WMD 42.55, 95% CI -1.69 -86.79)	Yes
			Success of the first breastfeeding		
			Breastfeeding problems such as breast engorgement, infant latch-on difficulties, sore nipples;		
			Breast milk maturation; Changes in infant physiological parameters during and after skin-to-skin contact (Additional outcomes considered-see reference)		
Ahmed & Sands [56]	8 (1999-2008)	Breast feeding interventions inc kangaroo care, peer counselling, in home breast milk measurement, post discharge lactation support	Duration	Kangaroo care, peer counselling, in home breast milk measurement, and post discharge lactation support improved breast feeding outcomes	No
			Exclusivity	Maternal satisfaction improved with post discharge support	
			Maternal satisfaction	No impact on weight gain	
			Weight gain		
***Mental Health***					
Dennis & Hodnett [57]	9 (1966-2006)	Postnatal psychosocial and psychological interventions	Postpartum depression	Psychological and psychosocial interventions were effective in decreasing depressive symptomatology within the first year postpartum (RR 0.70, CI 0.6 to 0.81).	Yes
			Maternal mortality		
***Education & Support***					
Amorim Adegboye *et al.*[58]	6 (1994-2003)	Diet, exercise or both for weight reduction postpartum	Change in body weight (kg), % of women who returned to pre pregnancy weight or lost weight retained after childbirth, % of women who achieved healthy weight,	Both women who took part in a diet (1 trial, n=45, WMD -1.70 kg; 95% CI -2.08 to -1.32, z=8.73; p<0.00001), and women on a diet plus exercise programme (4 trials, n=169, WMD -2.89 kg; 95% CI -4.83 to -0.95; z=2.92; p<=0.00049), lost significantly more weight than women in the usual care	Yes
Corcoran & Pillai [59]	16 (1970-2004)	Repeat pregnancy prevention programmes, including education and counselling (majority hospital-based interventions) for teenagers	Rates of repeat pregnancy	The prevention programme saw a 50% reduction in the odds of repeat pregnancy when compared to comparison-control conditions at mean 19.13 mths (OR 0.474, 95% CI 0.322-0.695), but the effect dissipated by mean 31 mths.	Yes
Pinquart & Teubert [60]	142 (not given)	Parenting education with new parents	Parenting stress	Small effects on parenting, parental stress, child abuse, health promoting behaviour, cognitive, Social development, motor development, child mental health, parental mental health & couple adjustment	Yes
			Parenting quality		
			Health promoting behavior		
			Child abuse and neglect		
			Child development		
			Mental health of parents		
			Couple adjustment		
Vanderveen *et al.*[61]	25 (1980-2006)	Early parental intervention for premature infants-varied but all involved teaching/enhancing parents skills or involving parents in aspects of care	Primary outcome: neurodevelopment other outcomes discussed in subsequent paper(not specified in paper, available from authors)	12 studies: Higher mental performance scores at 12 months (WMD 5.57 95% CI 2.29-8.86 p=0.0009) and at 24 months (7 studies, WMD 7.59 95% CI 5.01-14.31 p=0.0003) and at 36 months (2 studies, WMD 9.66 95% CI 5.01-14.31 p=0.0001) but not at 5 yrs (3 studies p=0.24)	Yes

Acronyms used: USS=Ultrasound Scan; MD=mean difference; NTD=neural tube defect; CI=confidence interval; IOL=induction of labour; RR=relative risk; B/P=blood pressure; C/S=caesarean section; ICU=intensive care unit; LBW=low birth weight; SGA=small for gestational age; NICU=neonatal intensive care unit; PTD=preterm delivery; IUGR=intrauterine growth retardation; RCT=randomised controlled trial; Hb=haemoglobin; MA=meta analysis; WMD=weighted mean difference; APH=antepartum haemorrhage; SVB=spontaneous vaginal birth; PPH=postpartum haemorrhage; SCU=special care unit; OR=odds ratio; IVH=intraventricular haemorrhage; ARM=artificial rupture of membranes; EFM=electronic fetal monitoring; PND=postnatal depression.

### Findings -effective interventions

#### Pre conceptual

There were no high quality reviews that reported on effective interventions in the pre conceptual period.

### Antenatal

The majority of reviews reporting effective interventions were relevant to the antenatal period (n=20). Included reviews have been grouped into screening, supplementation, support, education and mental health.

#### Screening

Reviews (n=4) related to screening reported on interventions relating to ultrasound [26,27], lower genital tract infection screening [28] and the use of decision making aids [29]. Bricker *et al.*[26] conducted a large Health Technology Assessment review on the clinical and cost effectiveness and women’s views of USS. The review comprised of three systematic reviews on routine ultrasound in early pregnancy, routine ultrasound in late pregnancy and routine Doppler ultrasound in pregnancy which were published in the Cochrane database around the time of Bricker *et al.*[26] however, all have since been updated or revised in the Cochrane database, one of which has been included in this paper. The final conclusions of Bricker *et al.*[26] indicated that a two stage regimen of USS in pregnancy, one in early pregnancy (booking USS) and a second anomaly USS around 20 weeks, was recommended. Whitworth *et al.*[27] reviewed the use of ultrasound for fetal assessment in early pregnancy and concluded that it reduces failure to detect multiple pregnancy (RR 0.07 95% CI 0.03-0.17) and accuracy of gestational dating may reduce the number of inductions of labour for post term gestation (RR 0.59; 95% CI 0.42-0.83). The authors also reported there was no reduction in adverse outcomes or health service use by mothers or infants and long term follow up did not indicate detrimental effect on children’s physical or mental development. The impact of antenatal screening for lower genital tract infection for preventing preterm delivery was reviewed by Sangkomkamhang *et al*. [28]. The review included one large RCT (n=4155), which indicated that preterm birth before 37 weeks was significantly lower in a group of women randomised to a screening programme before 20 weeks’ gestation (RR 0.55; 95% CI 0.41-0.75). The review provides evidence to suggest there may be some benefit to introducing a universal screening programme for lower genital tract infection; however the results are based on the findings of one study. O’Connor *et al.*[29] conducted a review on the use of decision aids for people facing screening decisions. The meta analysis indicated that the use of decision aids, such as leaflets or DVD’s are better than usual care and resulted in: greater knowledge (MD 15.2 out of 100; 95%CI 11.7 to 18.7), perception of risk (RR 0.6; 95% CI 0.5 to 0.8), lower decisional conflict related to feeling uninformed (MD −8.3 of 100; 95% CI −11.9 to −4.8), lower decisional conflict related to personal values (MD −6.4; 95% CI −10.0 to −2.7), reduced the proportion of people who were passive in decision making (RR 0.6; 95% CI 0.5-0.8) and reduced the proportion of people who remained undecided post intervention (RR 0.5; 95% CI 0.3-0.8). Although the results suggest decision aids are effective, the effect size was not consistent across studies and only three of the included studies related directly to antenatal screening.

#### Supplementation

Eight reviews [30-37] considered supplementation during pregnancy including iron, micronutrients, folic acid, calcium and Long Chain-Poly Unsaturated Fatty Acids (LC-PUFA’s). Two reviews [30,31] focused on folic acid supplementation, both of which concurred that the risk of neural tube defect was significantly reduced with supplementation: Blencowe et al., [30]; 70% reduction; 95% CI 35-86 and Lumley et al., [31]; RR 0.28; 95% CI 0.13-0.58. Iron supplementation during pregnancy was reviewed by Pena-Rosas and Viteri [32] who included 49 trials relating to the prevention of iron deficiency or anaemia at term. The authors concluded that daily iron supplementation was associated with increased haemoglobin before birth (MD 6.00; 95% CI 2.75-9.25) and reduced risk of anaemia at term (RR 0.46; 95% CI 0.29- 0.72) based on meta analyses of high quality trials only. Shah *et al.*[33] reviewed multi-micronutrient supplementation on pregnancy outcomes and reported there was a reduction in the risk of low birth weight amongst women given micronutrient supplementation (12 studies, RR 0.81; 95% CI 0.73-0.91) and iron-folic acid supplementation (RR 0.83; 95% CI 0.74-0.93) compared to placebo. The mean birth weight was higher (11 studies; WMD 54g; 95% CI 36-72g) in infants born to mothers who had micronutrient supplementation compared to iron-folic acid supplementation (no difference with placebo).

Calcium supplementation was the focus of three reviews [34-36]. Hofmeyr *et al.*[34] reported a reduction in pre-eclampsia (RR 0.68; 95% CI 0.57-0.81) and fewer babies born <2500g (RR 0.83; 95% CI 0.71-0.98). However the benefits seen were from small trials and not observed in the largest trial included. Hofmeyr *et al.*[35] reported that with supplementation a reduction in blood pressure (RR 0.7; 95% CI 0.57-0.86), pre-eclampsia (RR 0.48; 95% CI 0.33-0.69) and maternal death/morbidity (RR 0.80; 95% CI 0.65-0.97) was noted and advocated research to investigate calcium supplementation at community level. The most recent review [36] conducted by several of the same authors as Hofymeyr *et al.*[34] on calcium supplementation concluded that there was a reduced risk of increased blood pressure (RR 0.65; 95% CI 0.53-0.81) and preeclampsia (RR 0.45; 95% CI 0.31-0.65). The effect was greatest for high risk women (RR 0.22; 95% CI 0.12-0.42) and women with low baseline calcium (RR 0.36; 95% CI 0.20-0.65). Maternal death or serious morbidity was reduced (RR 0.80; 95% CI 0.65-0.97) although this was mostly in low risk women and women with low calcium and there was no effect on preterm births, stillbirth or death before discharge. Horvath *et al.*[37] reviewed the effect of advising high-risk pregnant women to take LC-PUFA supplementation on a number of pregnancy outcomes. The authors found a significantly lower rate of PTD <34 wks (RR 0.39; 95% CI 0.18-0.84) although this result was based on two trials (n=291). There was no effect on duration of pregnancy, PTD <37 wks, infant birth weight or the occurrence of IUGR. Although significant, the authors concluded that there was not enough evidence to recommend routine use of LC-PUFA supplements by high-risk women and that further research involving larger sample sizes was needed.

#### Support

Three reviews [38-40] considered different types of supportive interventions for women during pregnancy. These ranged from using midwifery models of care to provision of emotional support to reduce the risk of preterm delivery or low birth weight infants. Hatem *et al.*[38] reviewed midwife led models of care versus other models of care and concluded that the majority of women should be offered midwifery led care. Women who had midwife led models of care were less likely to experience antenatal hospitalisation (RR 0.90; 95% CI 0.81-0.99), use of regional analgesia (RR 0.81; 95% CI 0.73-0.91), episiotomy (RR 0.82; 95% CI 0.77-0.88) and instrumental delivery (RR 0.86; 95% CI 0.78 to 0.96) and were more likely to experience no intrapartum analgesia/anaesthesia (RR 1.16; 95% CI 1.05-1.29), vaginal delivery (RR 1.04; 95% CI 1.02-1.06), to feel in control during childbirth (RR 1.74; 95% CI 1.32-2.30), attendance at birth by a known midwife (RR 7.84; 95% CI 4.15-14.81) and initiate breastfeeding (RR 1.35; 95% CI 1.03 to 1.76). In addition, women who were randomised to receive midwife-led care were less likely to experience fetal loss before 24 weeks’ gestation (RR 0.79; 95% CI 0.65-0.97). There was no difference between groups for birth by caesarean section (RR 0.96; 95% CI 0.87-1.06) and no statistically significant differences in fetal loss/neonatal death of at least 24 weeks (RR 1.01; 95% CI 0.67-1.53) or fetal/neonatal death overall (RR 0.83; 95% CI 0.70-1.00) and their babies were more likely to have a shorter length of hospital stay (mean difference in days: -2.00; 95% CI −2.15 to −1.85). Hodnett & Fredericks [39] assessed the value of emotional support to women who were judged, by a health professional, to be at increased risk of preterm delivery or having a low birth weight baby. No significant effect was detected for either outcome, however, women receiving support interventions were significantly less likely to undergo a caesarean section (RR 0.88; 95% CI 0.79-0.98) and were more likely to terminate their pregnancy (RR 2.96; 95% CI 1.42-6.17). There was also a trend towards improvement in maternal psychosocial outcomes although this was not significant. Denis & Kingston [40] reviewed the effect of telephone support during pregnancy and early postpartum period specifically on smoking, preterm birth, low birth weight, breast feeding and postpartum depression. The authors report a positive effect on breast feeding (3 trials; n=618; RR=1.18; 95% CI 1.05-1.33), low birth weight (3 trials; n=2,027; RR=0.78; 95% CI 0.63-0.97) and postpartum depression at 4 weeks (RR 0.24; 95% CI 0.06-1.00) and 8 weeks (RR 0.30; 95% CI 0.10-0.92), although all were from small numbers of trials and the finding on postpartum depression was from one pilot trial including 42 women.

#### Education

Educational interventions in the antenatal period were the focus of four systematic reviews [41-44] that considered education about pelvic floor muscle training (PFMT) and promotion of smoking cessation in pregnancy Lumley *et al.*[41] reviewed the effect of interventions for promoting smoking cessation and included 72 studies of which 56 were RCT’s. Interventions to encourage cessation of smoking had a significant effect on the number of women smoking; 6 out of every 100 stopped, and a reduction in the number of cigarettes smoked by women was also evident. There was a significant reduction of smoking in late pregnancy (RR 0.94; 95% CI 0.93-0.96), reduction in LBW (RR 0.83; 95% CI 0.73 -0.95), preterm birth (RR 0.86; 95% CI 0.74-0.98) and an increase in mean birth weight (53.91g; 95% CI 10.44g - 95.38g). Naughton *et al.*, [42] reviewed the use of self help interventions for smoking and reported greater likelihood of quitting compared to usual care (13.2% v 4.9%; OR 1.83; 95% CI 1.23-2.73). The cost effectiveness of this method was also emphasised, however, further research is necessary to determine the intensity level of the intervention to maximise effectiveness. Hay-Smith *et al.*[43] and Lemos *et al.*[44] reviewed pelvic floor muscle training and concluded that for primigravida women PFMT was effective. Hay-Smith *et al.*[43] reported that women without prior incontinence were less likely to report incontinence in late pregnancy (RR 0.44; 95% CI 0.30-0.65) and up to 6 months postpartum (RR 0.71; 95% CI 0.52-0.97) similar to Lemos *et al.*[44] who reported significantly reduced development of urinary incontinence from 6 weeks to 3 months after delivery (OR 0.45; 95% CI 0.3-0.66; 4x RCT; n=675). Pregnant women with persistent incontinence 3 months after delivery and received PMFT were less likely to report urinary incontinence at 12 months post delivery (RR 0.79; 95% CI 0.70-0.90) and less likely to report faecal incontinence at 12 months (RR 0.52; 95%CI 0.31-0.87) [43].

#### Mental health

One review by Dennis & Creedy [45] considered interventions to prevent postnatal depression and all but one involved an intervention from a health professional. The authors reported that preliminary evidence suggests that intensive postnatal nursing home visits with at risk mothers assisted prevention of postpartum depression (RR 0.67; 95%CI 0.51-0.89).

### Intranatal

Eligible systematic reviews relevant to the intranatal period yielded the smallest number in comparison to either the antenatal or postnatal periods. Five reviews [46-50] were included in this section and considered either clinical care during labour/delivery or the birthing environment.

#### Clinical care

Cluett & Burns [46] reviewed immersion in water for labour or birth (n=11) and reported from a meta analysis of 6 RCT’s. There was evidence to indicate that immersion in water for the first stage of labour significantly reduced the rate of epidural, spinal, paracervical analgesia and anaesthetic analgesia (478/1254 versus 529/1245; OR 0.82; 95% CI 0.70-0.98; p 0.025). However further research is required on other outcomes where there was no difference identified including assisted vaginal deliveries, C/S, perineal trauma, maternal infection, Apgar score < 7 at 5 mins, neonatal unit admissions or neonatal infection rates. Rabe *et al.*[47] reviewed delayed umbilical cord clamping and indicated from a meta analysis that there are benefits for both term and preterm infants. A delay of 30–120 seconds of cord clamping reduced the need for transfusions (RR 2.01; 95% CI 1.24-3.27, p=0.0049) and intraventricular haemorrhage (RR 1.74; 95% CI 1.08-2.81, p=0.022) in infants born <37 weeks [47]. Although the short term benefits are clear, further longitudinal work is needed to clarify the long term benefits.

#### Environment

The birth setting was the subject of four reviews although all were on different aspects. Hodnett *et al.*[48] reviewed the evidence regarding alternative versus conventional institutional settings for birth, which did not include any trials conducted in free standing birth centres. The review reported that for women allocated to the intervention (alternative setting) there was a significant increased likelihood of no analgesia/anaesthesia (RR 1.17; 95% CI 1.01-1.35), spontaneous vaginal delivery (RR 1.04; 95% CI 1.02-1.06), very positive views of care (RR 1.96; 95% CI 1.78-2.15), breastfeeding rates at 6–8 weeks (RR 1.04; 95% CI 1.02-1.06) and decreased episiotomy rate (RR 0.83; 95% CI 0.77-0.90). There was no effect on serious perinatal or maternal morbidity or mortality. Continuous support during childbirth was reviewed by Hodnett *et al.*[49]. The intervention involved one to one support during labour and found increased likelihood of shorter labour (WMD −0.43 hours; 95% CI −0.83 to −0.04), spontaneous vaginal delivery (RR 1.07; 95% CI 1.04 to 1.12) and were less likely to have intrapartum analgesia (RR 0.89; 95% CI 0.82- 0.96) or report dissatisfaction with childbirth experience (RR 0.73; 95% CI 0.65- 0.83). The authors only reported on outcomes where at least four trials were included in the meta analysis and highlighted that, generally, continuous intrapartum support was associated with greater benefits when it was not a member of hospital staff, when it began in early labour and in settings where epidural was not routinely available. Hodnett *et al.*[49] concluded that continuous support should be the norm rather than the exception for all women and further research is required as to the effectiveness of doula or lay support.

One review considered interventions aimed at reducing caesarean section rates [50]. Chaillet & Dumont [50] reported from a meta analysis that regular audit, detailed feedback regarding aspects of caesarean section performance (responsibility for decision making, rates, review of cases in clinical practice and multi faceted strategy approaches, such as development of guidelines, education of health professionals and women about vaginal birth after caesarean section (VBAC) were effective for reducing the caesarean section rate (RR 0.81; 95% CI 0.75-0.87). Details of relative risk for each type of strategy are included in Table 3.

### Postnatal

Eleven reviews [51-61] reporting on effective interventions related to the postnatal period. The reviews ranged across four areas: breast feeding; mental health; education and support.

#### Breast feeding

Reviews on this topic generally related to either support or promotion of breastfeeding. Britton *et al.*[51] reviewed the evidence in relation to support for breastfeeding mothers and key findings indicated that all forms of extra support for any breastfeeding (exclusive or partial) increased the duration of breastfeeding (RR 0.91; 95% CI 0.86-0.96) and the effect was greater for exclusive breastfeeding (RR 0.81; 95% CI 0.74-0.89). These findings were supported by Chung *et al.*[52] and Sikorski *et al.*[53]. Breastfeeding interventions included in both Britton *et al.*[51] and Chung *et al.*[52] involved formal or structured breastfeeding education, informal breastfeeding education or breastfeeding support either lay or professional. Chung *et al.*[52] from a meta analysis of 34 studies reported that breastfeeding interventions were effective in relation to increasing short term (1-3mths) and long-term (6-8mths) exclusive breastfeeding (RR 1.28; 95% CI 1.11-1.48 and RR 1.44; 95% CI 1.13-1.84) although statistically significant heterogeneity was noted for short term exclusive breast feeding (I^2^ =55%; p= 0.006). The authors also highlighted an increased rate (22%) of any (RR 1.22; 95%CI 1.08-1.37) and exclusive (RR 1.65; 95%CI 1.03-2.63) short term breastfeeding with interventions that included a component of lay support. Sikorski *et al.*[53] reviewed additional support versus standard care and concluded that additional professional support was more beneficial than standard care for duration of any breastfeeding (RR 0.89, 95% CI 0.81-0.97; 10xRCT; n=19,696) and additional lay support was effective in reducing the cessation of exclusive breastfeeding (RR 0.66; 95% CI 0.49-0.89; 5xRCT; n=2530). Effect sizes for interventions with an antenatal education element (RR 0.85; 95% CI 0.70-1.04) were not statistically significant, while those with a postnatal element alone were (RR 0.80; 95% CI 0.80-0.96). Four trials using WHO/UNICEF training showed significant benefit in prolonging exclusive breastfeeding (RR 0.70; 95% CI 0.53-0.93), but were highly heterogeneous. The authors highlight the need to assess support in different settings especially with low rates, conduct economic analyses and use qualitative research to explore specific elements of support. Dyson *et al.*[54] focused on breastfeeding initiation rates and indicated from a meta analysis of five studies (n=582) that breastfeeding education had a significant effect on increasing initiation rates (RR 1.57, 95% CI 1.15-2.15, p=0.005) compared to standard care in low income groups although substantial statistical heterogeneity was noted (I^2^=53.4%). Early skin to skin contact was reviewed by Moore *et al.*[55] who reported statistically significant effects of early skin to skin on breastfeeding at one to four months post birth (OR 1.82, 95% CI 1.08-3.07) and breastfeeding duration (WMD 42.55, 95% CI −1.69 -86.79). In this review, data from more than two trials were only available for a small number of outcomes (8/64). Ahmed & Sands [56] reviewed breast feeding interventions. While the authors were unable to conduct a meta analysis they found from individual trials, statistically significant results relating to kangaroo care, peer counselling, in home breast milk measurement, and post discharge lactation support for improving breast feeding outcomes.

#### Mental health

One review focused on improving maternal mental health and considered postnatal psychological and psychosocial interventions [57]. Dennis & Hodnett [57] reported that any psychosocial or psychological intervention compared to usual postpartum care was associated with a reduction in the likelihood of continued depression from their review of nine trials. Examples of psychosocial and psychological interventions reviewed included non-directive counselling, supportive interactions, delivered via telephone, home or clinic visits, or individual or group sessions in the postpartum period by a health professional or lay person, cognitive behavioural therapy and interpersonal psychotherapy.

#### Education and support

One review considered support for women in relation to weight reduction in the post partum period [58] focusing on the effect of diet or exercise or both for reducing weight after childbirth. They found that women who took part in a diet (1 trial; n=45; WMD −1.70 kg; 95% CI −2.08 to −1.32; z=8.73; p<0.00001), and women on a diet plus exercise programme (4 trials; n=169; WMD −2.89 kg; 95% CI −4.83 to −0.95; z=2.92; p<=0.00049), lost significantly more weight than women in the usual care. The authors also noted that there was no adverse effect on breastfeeding, although cautioned that further research is necessary to confirm this finding. Three reviews considered extra support for vulnerable groups of women in the form of home visiting or parenting interventions [59-61]. Corcoran & Pillai [59] reviewed rates in repeat pregnancy following the introduction of hospital-based programmes providing education and counselling to a sample of adolescent mothers. They found that although there was a 50% reduction in the odds of repeat pregnancy compared to comparison-control conditions at 19months (OR 0.474; 95% CI 0.322-0.695), the effect had dissipated by 31 months. All studies were US based and the majority conducted in low income groups (74%) and African Americans (60%). Two reviews focused on parenting interventions [60,61]. Pinquart and Teubert [60] reported small effects on parenting, parental stress, child abuse, health promoting behaviour, cognitive, social development, motor development, child mental health, parental mental health & couple adjustment from parenting education interventions. Vanderveen *et al.*[61] demonstrated an overall positive effect on neurodevelopment from early parental interventions (all involved teaching or enhancing parental skills) lasting up to 36 months. Meta analysis of twelve studies indicated higher cognitive scores at 12 months (WMD 5.57; 95% CI 2.29-8.86; p=0.0009), at 24 months (7 studies; WMD 7.59; 95% CI 5.01-14.31; p=0.0003) and at 36 months (2 studies; WMD 9.66; 95% CI 5.01-14.31; p=0.0001), but not at 5 yrs (3 studies p=0.24). The authors suggest further research is needed to clarify the most effective interventions and the long term effect.

### Logic model

**Figure 2:**
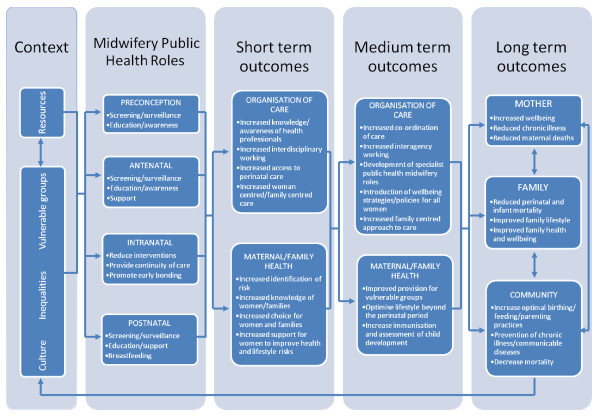
Summary Logic Model.

The parallel development of the logic model resulted in a summary model (Figure 2) provides a framework to visualise interventions across the perinatal period and the potential short, medium and long term impact on the health of women, their families and the community. Logic models display relationships between the core elements (context; inputs; outputs and outcomes) and the basic concept is to read from left to right, following a sequence of reasoning. An example of this is provision of education and information about screening in the antenatal period; an aspect of care where inequalities are known to occur [62]. The context in this example refers to the cultural, political, social circumstances in which the provision of screening is situated. Reading from left to right on the model indicates that the midwifery public health intervention is next so for example if a midwife provides information about antenatal screening for HIV (input), then uptake of screening may improve and at risk women will be identified earlier (outputs) and the effect will improve maternal and infant health during pregnancy. The medium and longer term outcomes are the resultant reduction in morbidity and or mortality in the local population.

The focus of this paper is the development of the public health role of the midwife based on effective interventions and highlighting the short, medium and long term effects that these interventions could bring about. Any intervention must be considered within the context in which it is to be delivered as inequalities, resources, culture and vulnerable groups can influence the choice of intervention to best suit the population of women being served. The second column represents the inputs or activities; these are the interventions which are intended to bring about the change in outcomes. In relation to public health and midwifery these are interventions that may impact on public health primarily through education, screening and support. The outputs are the products or the targets of the service delivered and can been seen in the boxes entitled organisation of care under short and medium term outcomes. While the logic model provides a visual outline of midwifery public health roles, using this approach facilitates understanding of how public health programs can be planned and subsequently evaluated. Conducting the data synthesis in tandem with developing the logic model has also highlighted where the gaps in knowledge are and identified areas where midwives could potentially have a much greater role and subsequent impact on public health.

## Discussion

This paper sought to report on systematic reviews providing high quality evidence of effective interventions, in essence the ‘cream of the crop’. Reviews reporting on effective interventions were those which presented a statistically significant meta analysis or where the intervention was supported by a generally positive trend of results when a meta analysis was not possible to ensure the recommendations of the paper are based on strong evidence of good quality. There were a number of reviews included which presented statistically significant positive findings. However, in some cases these were limited by small numbers of participants or small numbers of trials included in the review. As a result of conducting the review and analyzing eligible systematic review evidence, three key areas for future consideration were identified including: recommendation and implementation of effective evidence; gaps in knowledge and developing the role of the midwife in public health which are discussed further in the following sections.

### Recommendation and implementation of effective evidence

It is clear from this review of effective interventions, there are areas where evidence has been incorporated into guidelines and thus recommended for implementation into routine practice. However, it has also highlighted many areas where it has not. There has been extensive debate and commentary in the literature about knowledge transfer and translation of knowledge into practice, however, this paper confirms that despite the existence of good quality evidence, the gap remains. From this review, several effective interventions were identified, which are already recommended as routine practice, for example education about folic acid supplementation and pelvic floor muscle training to prevent or reduce the risk of urinary incontinence are advocated by current practice guidelines in the UK [63] and further afield [64,65]. However, to evaluate fully the extent to which guidelines have been applied it is essential to audit practice in order to provide evidence for knowledge transfer. To encourage implementation of NICE guidelines, audit support tools have been developed by NICE on antenatal care or diabetes in pregnancy for use at local level. Effective interventions were also identified which could easily be implemented by a midwife and could potentially impact on public health, such as education programs for parents of preterm infants and implementation of specific strategies to reduce caesarean section rates. Although there is recognition by health professionals these areas are important, this review provides definitive evidence and examples from systematic reviews, of interventions that are effective. Further consideration needs to be given to how to translate these effective interventions into practice using appropriate channels which are effective to facilitate knowledge transfer. These may include stronger collaborations between clinicians and academics and increasing the exposure students have to systematic reviews in education curricula at undergraduate level. Other effective interventions have been implemented on an ad hoc basis for example additional lay or professional support for breast feeding women and strategies to reduce caesarean section rates, which need to be included specifically in policy and strategy documents to ensure widespread implementation and thus contribute to an evidence based public health agenda to improve the health of women and families. Although this paper has focused on reporting effective interventions it is also important to take cognisance of those interventions that are not effective i.e. those which do not work and sometimes are deeply embedded into practice, for example, routine antenatal CTG for fetal assessment [20]. It was not possible to discuss reviews that demonstrated no effect within this current paper, however, Table 2 provides summary details of the areas where this was the case.

#### Gaps in knowledge

The review identified many gaps in systematic review literature relating to core midwifery practice, which potentially could impact on public health population goals. The UK Department of Health, Public Health Strategy [66] emphasizes the importance of improving maternal health and the subsequent impact on reducing infant mortality and premature births and yet this review identified limited systematic review evidence to support the implementation of midwifery interventions that could impact on perinatal morbidity and mortality. The review also highlighted it was difficult to accurately assess the potential public health impact in terms of effectiveness as some interventions were not well evaluated, evidenced by the large number of inconclusive reviews and reviews demonstrating no effect. The review of reviews identified some interventions that were effective but were limited in terms of methodological quality of included studies, for example, small numbers and design flaws, thus demonstrating the need for robust research and evaluation. One example of this is systematic review evidence in relation to weight management or obesity; a topic of growing concern to maternity care providers and yet the evidence from systematic reviews is limited in terms of quality. The systematic reviews included in the original review generally indicated that additional support related to diet or exercise for women in the postnatal period was effective, however, only one review was of a high quality. Another example of this is the evidence around home visiting for vulnerable groups of women in the postnatal period. While a significant body of research, including longitudinal studies has been published on parenting interventions indicating generally positive effects [67,68] the evidence from this current systematic review of reviews is mixed. Current early years governmental policy in the UK focuses on giving children the best start in life and various interventions have been, or are currently being rolled out, for example, SureStart and the Family Nurse Partnership, however the longer term impact on women and families remains to be seen. Logic models highlight the causal linkage between inputs, outputs and outcomes (24). This is illustrated very clearly in relation to support for parents in the form of parenting interventions (input) which can result in the short term outcome of increasing support for women to improve health and lifestyle; optimize lifestyle and child development beyond the immediate perinatal period (medium term) and in the long term improve family health and wellbeing for this generation and those to come.

#### Developing the role of the midwife in public health

In order for midwives to utilise their potential in relation to public health it is important not only to consider the interventions that could be implemented but also take cognisance of wider strategies and policy relating to public health. The logic model (Figure 2), which was developed as a parallel process to the review, provides an overarching framework that should be used by midwives to visualise their contribution to public health. The model illustrates possible future roles but also facilitates recognition of the current contribution of midwives to improving the health of women and their families as part of their core role. An example of this is how vulnerable women (either social or medical) could be identified in the antenatal period by midwives and a supportive or educational intervention implemented which would result in improved outcomes in the short term i.e. reduced pre term birth or improved birth weight. A medium term outcome of this intervention would focus on optimising lifestyle beyond the perinatal period for example collaborating with health visiting services to provide education and support that would potentially have a longer term outcome of improved family health and well being. The review did not identify any systematic reviews which specifically focused on interventions relating to midwifery public health roles, highlighting a gap in review evidence. Biro [69] suggests it may be challenging for midwives to think beyond individual women but ultimately necessary in order to meet the challenge of public health to improve population health. Reframing routine midwifery activities in a public health context, identifying midwives as public practitioners and building on existing activities, such as collaboration, organisation of care and interagency working are essential to clearly define the relationship between midwifery and public health. An earlier, wider review on health-led parenting interventions in pregnancy and the first three years of life [8] suggested that many interventions, particularly in relation to supporting parenting, could be provided as part of routine care and that although the optimal time to start programmes was not clear, there was some consensus that those initiated in the antenatal period were more effective. Development of the public health role of the midwife will also require strategic thinking and support from planners and commissioners of maternity services to ensure that midwives can influence policy and effectively implement public health strategies. This will involve dedicating time and resources to develop local policies, providing training for midwives and building good relationships with other healthcare disciplines to work together.

#### Limitations

There are a number of methodological challenges in using systematic review evidence which must be taken into account. It is difficult to summarise the evidence from systematic reviews as often there is significant diversity between interventions included in individual reviews or outcome measures used. In addition the results presented may be inconsistent between reviews or inconclusive, however, Smith *et al.*[14] suggest the strength of systematic reviews of reviews is that the best quality reviews can be highlighted in a single document. Systematic reviews are generally limited to published work and thus may be subject to publication bias. In addition, more recent, potentially conflicting, research may be available since the review was published or there may be effective interventions that have not been evaluated in a systematic review. A recent Cochrane overview of systematic reviews [70] highlighted that such reviews provide an accessible summary on the totality of the evidence in the area and minimised the need for referral to individual reviews, however suggested that readers may wish to do so for specific details. This review was similar in that it covered a broad scope of the evidence in relation to public health, providing a strategic overview while also providing a valuable resource for those who wish to consult individual reviews for additional specific details. In this paper, only high quality reviews (based on level of included evidence and methodology of review) reporting on effective interventions were included. While this provides reassurance regarding review findings, in that the conclusions are based on top level evidence, some interventions demonstrating effect may have been excluded because the review itself did not meet either the quality or level of evidence criteria for inclusion. In most cases this relates to areas worthy of future investigation, which need more robust evaluations. The search strategy utilised in the review was specifically focused on the public health role of the midwife and therefore incorporated key terms relative to key areas. However in doing so, some postnatal interventions, which extend beyond the role of the midwife, for example, parenting interventions that continue into early childhood may not have been included. In addition, due to the inclusion and exclusion criteria applied, it is possible that extensive broad reviews on particular topics have been excluded from this review due to the nature of evidence included within them, for example, the NICE Guideline on Antenatal and Postnatal Mental Health [9]. However, it is recognised these are valuable resources and contribute to wider understanding on specific subjects.

## Conclusions

This paper has reported on high quality effective interventions identified from a larger systematic review on public health interventions that could be delivered primarily by midwives or maternity care providers. From the effective interventions identified it is clear that while some have been recommended for implementation into routine practice, others have not. This highlights the continuing gap between evidence and practice and the need for professionals and researchers to work better together to ensure specific interventions that are effective, are translated into practice and subsequently audited to provide evidence of knowledge translation. The public health role of the midwives has not been well researched or reviewed and the impact of everyday midwifery practice on longer term, holistic maternal and family well-being outcomes is poorly articulated in review literature. A shift in research, policy and practice is needed to fully articulate the public health role of the midwife. This systematic review of systematic reviews identifies a number of effective interventions that provide a useful starting point on which to build future practice. The logic model demonstrates the need to fill in major gaps in our knowledge on effective interventions to achieve both short and long term public health benefits for women and their families. Such benefits will remain elusive without investment in a collaborative, strategic approach to the role of public health in midwifery.

## Competing interests

The authors declare that they have no competing interest.

## Advisory group members

*Ms Liz Bannon*, Senior Midwife, & Co Director of Maternity Services, Social Services, Family & Child Care Belfast Health and Social Care Trust, Belfast, Northern Ireland; *Professor Debra Bick*, Professor of Evidence Based Midwifery Practice, Kings College London, England; *Dr Helen Cheyne*, Nursing, Midwifery & Allied Professions Research Unit, University of Stirling, Scotland; *Professor Mike Clarke*, then Professor of Clinical Epidemiology & Director of UK Cochrane Centre, now Professor/Director of MRC Methodology Hub, Queen’s University Belfast; *Ms Joanne Gluck*, Consumer Representative; *Professor Billie Hunter*, Professor of Midwifery, Swansea University, Wales; *Dr Dermot O’Riley*, Centre of Excellence for Public Health Northern Ireland, Queen’s University Belfast, Northern Ireland.

## Authors’ contributions

JM extracted and interpreted data and wrote the first draft of the manuscript. FL conducted the searches of the literature, extracted and interpreted data and assisted with the manuscript. FA extracted and interpreted data and assisted with the manuscript. All authors read and approved the final manuscript.

## Pre-publication history

The pre-publication history for this paper can be accessed here:

http://www.biomedcentral.com/1471-2458/12/955/prepub
